# Handgrip Strength Asymmetry in Female Basketball Players: The Critical Role of Arm Position and the Challenge of Statistical Power

**DOI:** 10.3390/sports13080279

**Published:** 2025-08-21

**Authors:** Vassilios Panoutsakopoulos, Antonella V. Schwarz, Evangelia Merkou, Stratos Savvoulidis, Mariana C. Kotzamanidou, Zacharias Papadakis

**Affiliations:** 1 Biomechanics Laboratory, School of Physical Education and Sport Science at Thessaloniki, Aristotle University of Thessaloniki, 54124 Thessaloniki, Greece; bpanouts@phed.auth.gr (V.P.);; 2 Human Performance Laboratory, Department of Health Sciences and Clinical Practice, College of Health Professions and Medical Sciences, Barry University, Miami Shores, FL 33161, USA; zpapadakis@barry.edu; 3 Faculty of Health Sciences, Metropolitan College of Thessaloniki, 54624 Thessaloniki, Greece; 4 Department of Coaching and Physical Education, School of Sport Sciences and Physical Education, Metropolitan College of Thessaloniki, 54624 Thessaloniki, Greece; 5 Institute of Occupational Science & Rehabilitation, Metropolitan College, 15125 Athens, Greece

**Keywords:** biomechanical analysis, muscular strength, arm preference, joint angle, 2D:4D, interlimb difference, injury prevention, athlete health monitoring, body fat, adolescence

## Abstract

**Background:** Handgrip strength asymmetry is a critical yet underexplored basketball component. While the digit ratio (2D:4D) is linked to strength, its interplay with age, body composition, and biomechanics is unclear. This study aimed to quantify these independent and interactive effects on asymmetry in female basketball players. **Methods:** Maximum handgrip strength was measured bilaterally in three arm postures in 26 adolescent and adult players. Linear Mixed Model with a random intercept tested the effects. **Results:** Omnibus tests revealed no statistically significant main effects or interactions for age group, lean body mass (LBM), or 2D:4D ratio. However, a planned contrast showed that asymmetry was significantly lower in an overhead arm posture compared to an extended arm posture (*p* = 0.035). A simulation-based power analysis determined the study was significantly underpowered (11.5%) to detect small-to-medium interaction effects. **Conclusions:** While biomechanical position subtly modulates strength asymmetry, the influence of age, lean mass, and digit ratio may be negligible or require substantially larger samples to detect. Individual differences, which accounted for 57% of the variance, appear to be the dominant drivers of handgrip asymmetry in this athletic cohort, highlighting the complexity of strength imbalances and the critical need for adequately powered research in this domain.

## 1. Introduction

Digit ratio, also known as 2D:4D, reflects the length ratio between the index finger (2D) and ring finger (4D). Evidence indicates this biologically stable trait is established prenatally, becoming fixed as early as the second trimester of pregnancy [1,2] and remaining stable across the lifespan [3,4]. Sex differences in 2D:4D ratios emerge early, with males showing lower values than females, a pattern attributed to prenatal androgen exposure, particularly via the fourth digit’s high density of androgen receptors [5]. In practical terms, males tend to have index fingers shorter than their ring fingers (2D < 4D), while females generally show equal or longer index fingers (2D ≥ 4D). This sexually dimorphic pattern underscores why 2D:4D serves as a biomarker for prenatal testosterone exposure, reflecting the relative androgenization during critical periods of fetal development [5]. As a key prenatal organizer, testosterone influences skeletal, neural, and physiological systems [1,6], potentially contributing to later-life variations in physical strength capabilities.

Given testosterone’s role in musculoskeletal development, the 2D:4D ratio has emerged as a potential biomarker for athletic performance traits [7,8,9,10], including strength [11,12]. A growing body of evidence suggests that individuals exhibiting lower 2D:4D ratios tend to demonstrate superior athletic performance capabilities [7,8,9,10]. Notably, in open-skill sports like basketball, Dyer et al. [7] found that female athletes with lower 2D:4D ratios exhibited superior game performance metrics and were more likely to secure starting positions. These outcomes can probably be attributed to testosterone’s neuromuscular effects. However, these associations are not always consistent across studies, suggesting that the influence of prenatal androgenization may be complex and moderated by other biological or environmental factors. Furthermore, another factor relating to 2D:4D that can possibly affect the evaluation of the above-mentioned outcomes is the assessment instrument. For example, a recent study reported that the size of the hand could reduce the reliability of the handgrip strength measure due to the dimensions and the shape of the handgrip dynamometer [13].

Testosterone’s organizational effects on motor neuron development and muscle fiber composition [14,15] may predispose individuals to asymmetrical strength patterns. Prenatal androgen exposure could enhance dominant-hand neuromuscular efficiency [16], potentially manifesting as grip strength disparities. However, whether these effects extend to handgrip strength asymmetry, a critical factor in basketball’s unilateral tasks (e.g., shooting), remains unclear.

Handgrip strength asymmetry may further depend on modifiable factors, like lean mass, a proxy for muscle capacity, and arm position, which alter biomechanical leverage [17]. While lean body mass (LBM) reflects overall muscle potential [18,19], its distribution between limbs may interact with 2D:4D to amplify asymmetries. Importantly, LBM is not only a marker for general strength, but also a critical component of basketball-specific fitness [20,21]. While traditional anthropometric, body composition, and strength variables are poor predictors of jump performance [22], vertical jump testing is still the main evaluation tool to identify meaningful strength asymmetries in basketball players [23,24,25,26,27]. On the other hand, the handgrip strength is commonly used to acquire information regarding strength output asymmetries for the upper extremities [28,29,30,31].

Crucially, the biomechanical context in which strength is tested, specifically arm position, is a critical determinant of force output [32] that can either mask or exaggerate underlying imbalances. For example, overhead grips (e.g., during rebound) recruit different muscle groups (i.e., the biceps brachii, the adductor muscles of the shoulder, the wrist, and the finger flexors [33]) than neutral position dibbling (where flexor carpi radialis and triceps brachii are the main actors [34]), potentially exaggerating or masking innate strength disparities. Other basketball skills, i.e., ball holding, passing, and shooting, are essentially based on arm muscle strength, with hand grip strength testing, along with anthropometric measures, being of importance for talent identification and performance evaluation [35,36]. In detail, a crucial factor examined in handgrip strength testing is the relative angular position of the upper extremity joints [37,38,39,40,41,42,43,44,45,46,47]. Different combinations of shoulder–elbow–wrist position resulted in different strength outcomes [39,40,41]. Previous research suggests that, in the case of a fully extended elbow, the highest mean grip strength is observed when the shoulder is at 180 deg of flexion [44,46]. In college women, the 90-degree-flexed elbow leads to greater hand grip strength compared to a straight arm condition [42]. However, the greater sex difference was observed at the condition with 90 deg elbow flexion when the shoulder is at 0 deg flexion [38]. Finally, the wrist set at a 30 deg extension was found to be the condition with the largest handgrip strength compared to other forearm postures [45]. Nevertheless, although a flexed wrist results in lower strength output, the muscle activation remains unchanged [43]. Despite basketball’s reliance on unilateral actions, few studies have examined handgrip strength asymmetry in female basketball players [35,48,49,50], and, to the best of our knowledge, none have investigated how these critical biomechanical factors (e.g., arm position) interact with innate traits like 2D:4D to shape handgrip asymmetry in female athletes.

The developmental stage of the athlete introduces another layer of complexity. In adolescent females, varying pubertal maturation rates may obscure the relationship between 2D:4D and strength asymmetry, as transient hormonal changes temporarily override prenatal influences [51]. Additionally, ongoing bone and muscle development during this stage [52] may impair neuromuscular control, contributing to more pronounced, yet potentially temporary, strength asymmetries. In contrast, adult female athletes are nearing peak muscle mass [53], meaning any observed asymmetries are more likely attributed to training habits or prior injuries rather than growth-related imbalances. Further, their more stable neuromuscular control likely reduces natural fluctuations in asymmetry, making persistent imbalances more reflective of sport-specific adaptations or underlying biomechanical disparities. Nevertheless, asymmetries in sports result from limb dominance and can be enlarged because of the submission in long-term systematic training [54]. Moreover, the relative literature suggests that inter-limb asymmetry in force application is related to musculoskeletal injury [55]. Previous studies have suggested that asymmetry values of about 15% could be considered as an initial indicator of significant asymmetry and that this is associated with a higher risk of injury [56,57,58,59], but this percentage is not universally supported by the literature [60].

While factors like 2D:4D ratio and LBM have been linked to athletic potential, their relationship with handgrip symmetry, particularly when considering the powerful influence of arm position and developmental stage, remains largely unexplored. Therefore, the aim of this study was to investigate the potential independent and interactive contributions of 2D:4D ratio, LBM, and arm position to handgrip strength asymmetry in adolescent versus adult female basketball players. This work sought to untangle this multifactorial problem while rigorously assessing the statistical power required to confidently detect such complex effects [61,62]. Based on the reviewed literature, it was hypothesized that a lower 2D:4D ratio and higher LBM would predict greater handgrip strength asymmetry. Furthermore, we predicted that the magnitude of this asymmetry would be significantly modulated by the biomechanical arm position during testing and that these relationships might differ between adolescent and adult athletes due to developmental factors.

## 2. Materials and Methods

### 2.1. Participants

A local semi-professional women’s basketball club competing in the regional league in Northern Greece and its affiliated U16 female youth basketball team served as a convenient sample. The adult group comprised 13 adult female (22.76 ± 7.42 years, 1.72 ± 0.07 m, 68.55 ± 10.64 kg, and 23.0 ± 2.79 kg/m^2^ for age, body height, body mass, and body mass index, respectively) players and the U16 adolescent group of 13 female (13.85 ± 1.07 years, 1.68 ± 0.06 m, 62.22 ± 10.61 kg, and 21.92 ± 2.57 kg/m^2^ for age, body height, body mass, and body mass index, respectively) non-professional basketball players.

Participation was allowed if the inclusion criteria were met, namely the involvement in systematic basketball training at least four times weekly, with a record of 90% participation in the training program. The exclusion criteria were the existence of orthopaedical and/or neurological impairments, a musculoskeletal injury within a period of 3 months before the testing, and the absence of a good health condition (namely the presence of respiratory, gastrointestinal, cardiovascular, neurological, or other disorders). Participation was granted with the provision of signed consent or informed parental/guardian consent in the case of the U16 players. The study was conducted in accordance with the Declaration of Helsinki, and after acquiring ethical approval from the Research Ethics Committee of the School of Physical Education and Sport Science at Thessaloniki, Aristotle University of Thessaloniki, Greece (approval code: 262/2025, 11 May 2025).

### 2.2. Experimental Procedures

#### 2.2.1. Anthropometric and Body Composition Measures

A cross-sectional study was conducted to investigate the hypothesis of the study. All testing was carried out in the Laboratory during the morning hours in ambient conditions. The testing was conducted at the beginning of the pre-season training period.

At first, body height and mass were measured using a Delmac PS400L scale (Delmac Instruments S.A., Athens, Greece) and a Seca 220 stadiometer (Seca Deutschland, Hamburg, Germany). Further anthropometric data were collected as selected skinfolds of the upper arm (biceps and triceps brachii area), the torso (abdominal, suprailiac, and subscapular area), and the lower extremities (frontal area of the thigh and at the calf muscles) were measured using a John Bull caliper (British Indicators LTD, Weybridge, Surrey, UK). Percentage body fat (%BF) and LBM were calculated following well-established [63] methods for adults [64] and adolescents [65].

#### 2.2.2. Handgrip Strength Protocol

Participants then executed a typical warm-up routine of 5 min low-intensity cycling on a cycle ergometer (817E Monark Exercise Cycle, Exercise AB, Vansbro, Sweden), followed by 5 min of dynamic exercises and stretching for the shoulder and the upper arm muscles. A couple of familiarization trials with the experimental setup were allowed.

**Figure 1:**
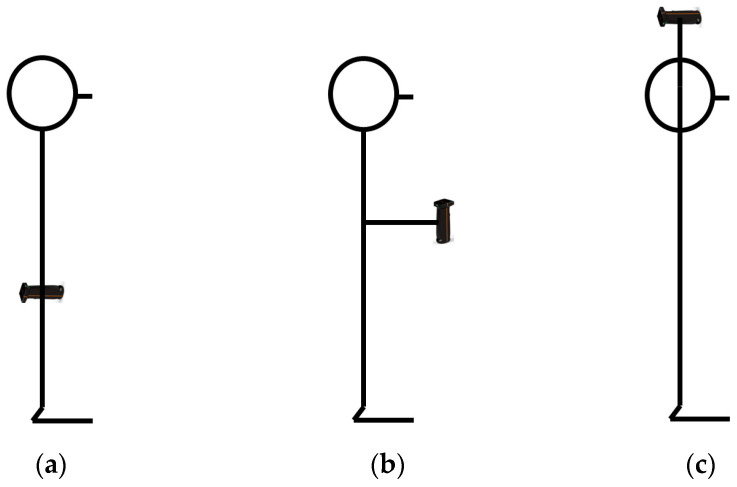
The arm postures examined for the handgrip strength tests: (**a**) AP1 (shoulder at 0 deg flexion, elbow at 180 deg extension, and wrist at neutral position); (**b**) AP2 (shoulder at 0 deg flexion, elbow at 90 deg flexion, and wrist at neutral position); (**c**) AP3 (shoulder at 180 deg flexion, elbow at 180 deg extension, and wrist at neutral position).

The testing procedure consisted of 3 maximum 5 s maximum handgrip isometric voluntary contractions (MIVC), with a 15 s rest interval. A K-grip v.2 (KINVENT Biomecanique, Montpellier, France) handgrip dynamometer was used to measure the force applied bilaterally in three distinct arm postures (Figure 1):

- Shoulder at 0 deg flexion, elbow at 180 deg extension, and wrist at neutral position with the palm facing towards the body (AP1);
- Shoulder at 0 deg flexion, elbow at 90 deg flexion, and wrist at neutral position with the palm facing inwards (AP2);
- Shoulder at 180 deg flexion, elbow at 180 deg extension, and wrist at neutral position with the palm facing inwards (AP3).

The instructions provided to the participants were the following: “Place your grip firmly on the dynamometer—After the ‘start’ signal, squeeze the dynamometer as hard and as fast as possible—release your grip after the ‘stop’ signal”. Verbal encouragement was not provided during the trials [66].

The tests were executed in the standing position. At the sagittal plane, a 2.5 m × 2.5 m reference frame was positioned to provide control for the verticality of the body and the orientation of the upper extremity segments. The tests were performed in random order regarding the arm posture and arm dominance. The dominant arm was determined by a questionnaire incorporating informative type data regarding the preference in use of the upper extremities in certain sport-related activities, i.e., the preferred arm to shoot, pass, and dribble the basketball [67].

A Samsung Galaxy A04s smartphone (Samsung, Incheon, Republic of Korea) recorded wirelessly the MVIC data (sampling frequency: 75 Hz). The peak recorded MVIC value, as well as the average MVIC value of the three trials were extracted using the K-Physio v. 2.20.3.1 application (Kinvent Biomecanique, Montpellier, France).

#### 2.2.3. Digit Ratio Measurement and Asymmetry Calculation

**Figure 2:**
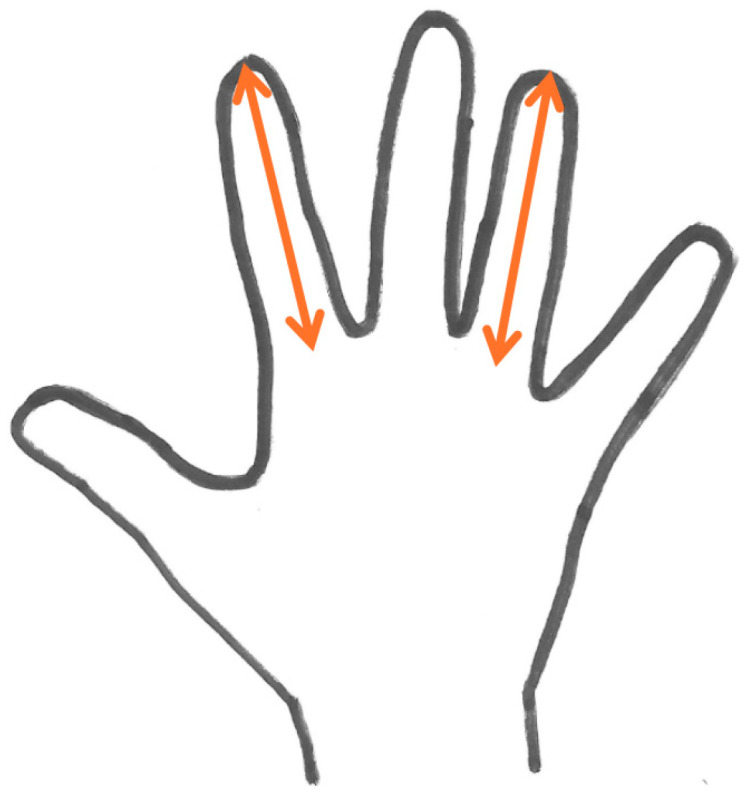
Depiction of the length of the index (2D) and ring finger (4D) measurement.

The 2D and 4D lengths were measured as depicted in Figure 2 using the Kinovea 2023.1.1 software (copyright © 2023 Joan Charmant and contributors). The distance from the middle of the base to the tip of the fingers was used to calculate the 2D:4D ratio. To acquire the data for the hand dimensions, a Konica Minolta Bizhub 223 Scanner (Konica Minolta, Inc., Tokyo, Japan) connected online with a personal computer was used. Participants sat at an adjustable seat in order to place both their hands firmly on the surface of the scanner with their hands aligned with the forearms, which were parallel to the ground (elbows flexed 90 deg).

The magnitude of the inter-limb asymmetry in the MVIC parameters was measured using the symmetry angle [68] (Equation (1)):

(1) $$\mathrm{symmetry angle}=\frac{\left(45{}^{\circ}-\arctan\left(\frac{\mathrm{non}-\mathrm{dominant}\;\mathrm{arm}}{\mathrm{dominant}\;\mathrm{arm}}\right)\right)}{90{}^{\circ}}\times 100\%$$

In the case where the condition described in Equation (2) was evident:

(2) $$\left(45{}^{\circ}-\arctan\left(\frac{\mathrm{non}-\mathrm{dominant}\;\mathrm{arm}}{\mathrm{dominant}\;\mathrm{arm}}\right)\right)>90{}^{\circ}$$

then Equation (1) was replaced by Equation (3):

(3) $$\mathrm{symmetry angle}=\frac{\left(45{}^{\circ}-\arctan\left(\frac{\mathrm{non}-\mathrm{dominant}\;\mathrm{arm}}{\mathrm{dominant}\;\mathrm{arm}}\right)-180{}^{\circ}\right)}{90{}^{\circ}}\times 100\%$$

### 2.3. Statistical Analysis

All statistical analyses were conducted using Jamovi v.2.6 (The Jamovi Project, Sydney, Australia) via the GAMLj module (Version 2.4.8), which leverages the lme4 package in R (Version 4.4, The R Foundation for Statistical Computing, Vienna, Austria). An alpha level of *p* < 0.05 was established a priori for all tests of statistical significance.

Prior to the main analysis, a series of preliminary checks was performed. The intra-test reliability of the handgrip strength measurements was examined using an intraclass correlation coefficient (ICC) with a two-way mixed-effects model for absolute agreement, with values interpreted as poor (<0.5), moderate (0.5–0.75), good (0.75–0.9), or excellent (>0.9) [69]. Data distributions were checked for normality using the Shapiro–Wilk test (*p* > 0.05). To examine baseline differences between the adolescent and adult groups, independent sample *t*-tests were conducted. The magnitude of these differences was assessed using Cohen’s d, interpreted as small (*d* < 0.2), moderate (0.2 ≤ *d* < 0.8), and large (*d* ≥ 0.8) [70].

To address the primary research question, a Linear Mixed Model (LMM) was employed. This analytical approach was selected over traditional repeated-measures ANOVA for several critical reasons: its superior handling of the nested data structure (three arm position measurements nested within each participant), its robustness to violations of sphericity, and its capacity to incorporate continuous covariates and their interactions seamlessly [71,72,73]. The dependent variable for all analyses was the symmetry angle (%). The analysis was performed on a dataset of 78 observations from 26 unique participants. To mitigate multicollinearity and facilitate the interpretation of main effects, the continuous predictors, LBM and 2D:4D ratio, were grand-mean-centered prior to their inclusion in the model.

The model-building process proceeded with a focus on identifying the most parsimonious model that adequately explained the data while adhering to statistical assumptions. An initial, fully saturated factorial model (including all main effects and all possible two-, three-, and four-way interactions) was first considered. However, an inspection of this model’s diagnostic plots revealed a significant violation of the assumption of normality of residuals (Shapiro–Wilk test: *W* = 0.96, *p* = 0.024), rendering its parameter estimates unreliable. This violation, coupled with the model’s excessive complexity relative to the sample size (24 fixed effects parameters for 26 participants), indicated a high risk of overfitting. Consequently, a more theoretically driven and parsimonious model was specified as the final model for inference. This model included the following fixed effects: age group (adolescent vs. adult), arm position (AP1, AP2, AP3), centered LBM, centered 2D:4D ratio, and the primary two-way interaction of interest, age group and arm position. To account for the non-independence of observations and model individual differences in baseline asymmetry, a random intercept was included for each participant identification number (ID) [74].

Model parameters were estimated using Restricted Maximum Likelihood (REML), and *p*-values for the fixed effects were calculated using the Satterthwaite approximation [75]. The final selected model was rigorously checked for adherence to statistical assumptions, and the residuals were confirmed to be normally distributed (Shapiro–Wilk test: *W* = 0.98, *p* = 0.278) [76].

Finally, a comprehensive a priori power analysis was conducted. A preliminary calculation in G*Power v. 3.1.9.6 (Universität Kiel, Kiel, Germany), based on a repeated-measures ANOVA analog, indicated that a sample of 28 participants would be needed to detect a medium-sized interaction (*f* = 0.25) with 80% power. However, acknowledging the limitations of this approach for complex LMMs, a more robust, model-specific power analysis was performed using Monte Carlo simulation (1000 runs) in R with the simr package. This simulation was specified to detect a small-to-medium interaction effect (Cohen’s *f*^2^ ≈ 0.06) within the exact structure of the final LMM, using the variance components derived from the fitted model (PID variance = 6.18, residual variance = 4.59). This gold-standard analysis revealed that the current sample size of *n* = 26 yielded a statistical power of 11.5% (95% CI: 9.6%, 13.6%). A power curve analysis further indicated that to achieve the conventional 80% power, a sample of approximately 110 participants would be required [77].

## 3. Results

The baseline demographic and anthropometric characteristics of the participants are presented in Table 1. As expected, the adult group was significantly older than the adolescent group. There were no other significant baseline differences between the groups for any other measured variable.

A LMM was fitted to the data to quantify the independent and interactive effects on the symmetry angle. The inclusion of a random intercept for PID was strongly justified, with the ICC for the final model indicating that 57% of the total variance in the symmetry angle was attributable to stable, between-participant differences. The overall model, combining both fixed and random effects, explained a substantial portion of the total variance (Conditional *R*^2^ = 0.60), though the fixed effects alone accounted for a smaller, non-significant portion (Marginal *R*^2^ = 0.07).

The omnibus tests for the fixed effects revealed no statistically significant main effects or interactions at the conventional alpha level of 0.05. The main effect for arm position approached significance (*F*(2, 48.00) = 2.81, *p* = 0.070), as did the primary interaction of interest (age group × arm position; *F*(2, 48.00) = 1.81, *p* = 0.174). The complete results of the fixed effects omnibus tests are presented in Table 2.

**Figure 3:**
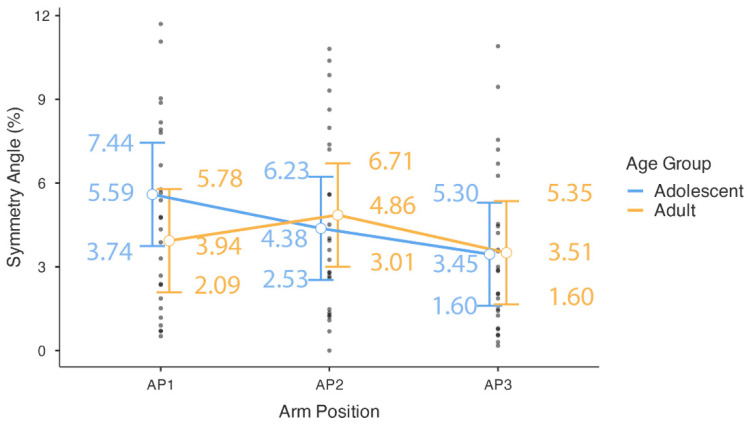
Symmetry angle by arm position and age group. The plot displays estimated marginal means with 95% confidence intervals. Individual observed scores for each participant are overlaid. Arm positions are defined as follows: AP1 (shoulder 0 deg, elbow 180 deg), AP2 (shoulder 0 deg, elbow 90 deg), and AP3 (shoulder 180 deg, elbow 180 deg).

The parameter estimates for the final model are detailed in Table 3. Although the omnibus test for the main effect of arm position was not significant, a planned contrast within the model revealed that the symmetry angle was significantly lower in the AP3 position (overhead) compared to the AP1 position (extended arm at the side of the body; estimate = −1.29, SE = 0.59, *t*(48.00) = −2.17, *p* = 0.035). No other pairwise comparisons within arm position were significant. Similarly, while the overall age group × arm position interaction was not significant, the specific contrast examining the differential effect of moving from AP1 to AP2 between age groups approached significance (Estimate = 2.14, SE = 1.19, *t*(48.00) = 1.80, *p* = 0.078). Given the non-significant omnibus tests, these specific parameter estimates should be interpreted with caution (Figure 3).

## 4. Discussion

The primary aim of this study was to investigate the independent and interactive contributions of age, digit ratio, LBM, and arm position to handgrip strength asymmetry in female basketball players. The central finding was a lack of statistically significant main or interactive effects for any of the theorized predictors in the omnibus tests. This outcome challenges the hypothesis that asymmetry is strongly dictated by a simple combination of innate traits, body composition, or developmental stage. Instead, the results imply a more nuanced reality where the biomechanical context of movement plays a subtle role, but the largest source of variance in strength asymmetry is attributable to stable, unmeasured differences between individual athletes.

The non-significant finding for the 2D:4D ratio, despite its theoretical link to prenatal testosterone and athletic performance [7,8,9,10], aligns with the notion that these associations can be inconsistent and complex. While prenatal androgens are known to be powerful physiological organizers [1,6], their specific influence on a nuanced outcome like strength asymmetry may be too subtle to detect without a very large sample, or it may be obscured by other factors. Similarly, the lack of an effect for the age group could be explained by the high intra-group variability introduced by transient developmental changes in adolescents [51,52] and highly specialized training adaptations in adults [53], effectively masking any clear between-group difference.

The non-significant contribution of LBM to asymmetry was also noteworthy. While LBM is a robust predictor of absolute strength [18], it may not directly influence asymmetry, which reflects relative rather than total strength differences between limbs. Athletes with greater LBM may have higher strength bilaterally, yet the proportional imbalance between dominant and non-dominant limbs could remain unchanged or even increase depending on sport-specific demands. This suggests that neuromuscular factors, such as inter-limb coordination, motor unit recruitment patterns, or habitual use differences, may play a more central role in asymmetry than gross muscle mass alone. Thus, while we hypothesized that LBM would contribute to asymmetry, the findings imply that functional adaptations related to task specificity may overshadow anatomical traits in predicting strength discrepancies.

In contrast, the subtle effect of arm posture was the only factor that approached statistical significance. The finding that asymmetry was significantly lower in the overhead position (AP3) compared to the extended arm position (AP1) supports the broad literature indicating that biomechanical context is a critical determinant of force output [37,38,39,40,41,42,43,44,45,46,47]. This suggests that while innate or anthropometric traits may not be strong predictors of asymmetry, the specific physical position in which an athlete performs a task can meaningfully alter the expression of strength imbalances. Despite the fact that basketball skills require a degree of bimanual dexterity [78], the strength output is found to be different between the dominant and the non-dominant upper arm [67,79], especially in young basketball players [28,80]. It is suggested that the dominant arm is stronger and more powerful in all types of muscle contraction because of its more frequent and higher-demand function to optimize performance in sport-specific tasks [81,82,83]. It should also be stressed that common strength-training practice might have an impact on this finding. In detail, it is recommended to gain strength for dribbling the ball using single-arm exercises/drills (an arm position similar to AP1 and AP2), whereas two-armed strength exercises are proposed to develop sport-specific strength for rebounding (arm position resembling AP3) and passing [84]. Thus, it seems logical that the observed asymmetries could result from limb dominance and be augmented by long-term systematic training and competition [54,85] in the sport of basketball. The trend toward a significant interaction contrast (*p* = 0.078) further hints that maturational status might influence how asymmetry manifests across different functional positions, a hypothesis that warrants future, better-powered investigation.

The results of this study must be interpreted within the context of its strengths and limitations. Strengths include the use of a Linear Mixed Model to appropriately handle the nested data structure and the implementation of a gold-standard Monte Carlo simulation to assess statistical power [71,72,73,74,75,76,86]. Paradoxically, the study’s primary strength, the transparent reporting of a gold-standard Monte Carlo simulation, is also what highlights its primary limitation. The study was severely underpowered (11.5%) to reliably detect the small-to-medium effects it set out to investigate. Therefore, we cannot conclude that the effects of 2D:4D or LBM are truly zero, only that they were not detectable in this sample. A secondary limitation may be the ecological validity of the tested arm postures relative to specific movements. Isometric laboratory tests, while reliable and controlled, may not accurately reflect the complex, dynamic, and variable nature of movements performed in real-world environmental settings [87,88,89,90,91,92,93,94,95]. Additionally, while this study incorporated 2D:4D as a proxy biomarker to account for anatomical variance, future research should directly examine the relationship between hand size and digit ratio to clarify their potential interaction and influence on grip strength assessments. The clear implication is that future research in this area is critically dependent on recruiting substantially larger samples (N > 110) to provide a definitive test of these complex interactions and to allow for replications of the findings [61,62,77].

Theoretically, these findings question the utility of relying solely on biological markers like digit ratio or LBM when studying strength asymmetry in athletes. While digit ratio is often cited as a proxy for prenatal androgen exposure and linked to athletic performance, its predictive power for nuanced motor traits like inter-limb strength discrepancies appears limited without extremely large sample sizes. Similarly, although LBM is a strong determinant of absolute strength, it does not appear to account for relative strength imbalances, emphasizing that neuromuscular and task-specific adaptations may play a more central role. This shifts the theoretical focus from static anatomical traits toward dynamic, functionally acquired characteristics, such as motor unit recruitment patterns, habitual movement use, and coordination.

Practically, the study offers important insights for coaches, athletic trainers, and strength and conditioning professionals. The effect of arm posture suggests that testing and training asymmetry in sport-relevant positions may provide more useful assessments and better targeted interventions. For example, bilateral overhead movements may better reflect symmetrical force generation, while unilateral or lower arm postures may highlight limb dominance. Furthermore, the lack of association between LBM and asymmetry implies that simply increasing muscle mass is unlikely to correct strength imbalances. Instead, unilateral training protocols, neuromuscular coordination drills, and sport-specific movement assessments may be more beneficial for managing or mitigating asymmetry.

## 5. Conclusions

Handgrip strength asymmetry in adolescent and adult female basketball players does not appear to be strongly predicted by common developmental or anthropometric markers such as age, 2D:4D ratio, or LBM. The phenomenon is dominated by high inter-individual variability, with biomechanical arm position acting as a subtle, yet significant, modulator of strength balance. The principal contribution of this study is the rigorous demonstration that research in this area is likely plagued by insufficient statistical power. Future investigations must prioritize large-scale, adequately powered designs to move beyond the study of individual differences and successfully identify the systematic factors that contribute to strength imbalances in athletic populations. These findings underscore the importance of considering functional context and sport-specific demands when assessing upper limb strength, as even small biomechanical differences can influence performance outcomes. Coaches are also advised to monitor upper-limb strength across various arm postures and to design training programs that enhance strength and control in sport-specific positions, ultimately contributing to improved performance and a reduced risk of injury.

## Figures and Tables

**Table 1:** Participant characteristics by age group (mean ± SD).

Variable	Adult (*n* = 13)	Adolescent (*n* = 13)	*t*-Statistic	*p*-Value	Cohen’s *d* [95% CI]
Age (years)	22.76 ± 7.42	13.85 ± 1.07	4.29	<0.001	1.68 [0.77, 2.57]
Body Height (m)	1.72 ± 0.07	1.68 ± 0.06	1.66	0.110	0.65 [−0.15, 1.44]
Body Mass (kg)	68.55 ± 10.64	62.22 ± 10.61	1.52	0.142	0.60 [−0.20, 1.38]
BMI (m/kg^2^)	23.00 ± 2.79	21.92 ± 2.57	1.02	0.316	0.40 [−0.38, 1.17]
Lean Body Mass (kg)	48.91 ± 7.09	47.21 ± 6.74	0.63	0.537	0.25 [−0.53, 1.01]
2D:4D Ratio	0.97 ± 0.02	0.97 ± 0.03	−0.29	0.771	−0.12 [−0.88, 0.66]
Symmetry Angle (%)					
Arm Position—AP1	4.02 ± 2.52	5.51 ± 3.93			
Arm Position—AP2	4.94 ± 3.83	4.30 ± 2.84			
Arm Position—AP3	3.59 ± 2.68	3.37 ± 3.28			

Note: The independent samples *t*-test was used for all comparisons with Ha μAdult ≠ μAdolescent. The degree of freedom for all tests is 24. CI: Confidence intervals; BMI: body mass index; 2D:4D ratio: the ratio of the length of the second digit (index finger) to the length of the fourth digit (ring finger) on a hand; AP1: shoulder at 0 deg flexion, elbow at 180 deg extension, and wrist at neutral position with the palm facing towards the body; AP2: shoulder at 0 deg flexion, elbow at 90 deg flexion, and wrist at neutral position with the palm facing inwards; AP3: shoulder at 180 deg flexion, elbow at 180 deg extension, and wrist at neutral position with the palm facing inwards.

**Table 2:** Fixed effects omnibus test results from the final linear mixed model.

	F	df	df (res)	*p*
Age Group	0.12	1	22.00	0.738
Arm Position	2.81	2	48.00	0.070
Lean body mass	0.27	1	22.00	0.606
2D:4D ratio	0.72	1	22.00	0.406
Age Group–Arm Position	1.81	2	48.00	0.174

Note: *F*-statistics and *p*-values are based on Satterthwaite’s approximation for degrees of freedom (df1, df2). 2D:4D ratio: the ratio of the length of the second digit (index finger) to the length of the fourth digit (ring finger) on a hand.

**Table 3:** Parameter estimates for the fixed effects of the final model.

				95% Confidence Intervals				
Names	Effect	Estimate	SE	Lower	Upper	df	t	*p*
(Intercept)	(Intercept)	4.29	0.54	3.20	5.37	22.00	7.87	<0.001
Age Group1	Adult–Adolescent	−0.37	1.10	−2.58	1.83	22.00	−0.34	0.738
Arm Position1	AP2–AP1	−0.15	0.59	−1.33	1.04	48.00	−0.25	0.804
Arm Position2	AP3–AP1	−1.29	0.59	−2.47	−0.10	48.00	−2.17	0.035 *
Lean body mass	Lean body mass	0.04	0.08	−0.12	0.21	22.00	0.52	0.606
D2:4D ratio	D2:4D ratio	−18.86	22.28	−63.32	25.60	22.00	−0.85	0.406
Age Group1 Arm Position1	(Adult–Adolescent) (AP2–AP1)	2.14	1.19	−0.23	4.51	48.00	1.80	0.078
Age Group1 Arm Position2	(Adult–Adolescent) (AP3–AP1)	1.71	1.19	−0.66	4.08	48.00	1.44	0.156

Note: An asterisk (*) denotes statistical significance at *p* < 0.05. Reference categories are Adolescent for the age group and AP1 for the arm position. 2D:4D ratio: the ratio of the length of the second digit (index finger) to the length of the fourth digit (ring finger) on a hand; AP1: shoulder at 0 deg flexion, elbow at 180 deg extension, and wrist at neutral position with the palm facing towards the body; AP2: shoulder at 0 deg flexion, elbow at 90 deg flexion, and wrist at neutral position with the palm facing inwards; AP3: shoulder at 180 deg flexion, elbow at 180 deg extension, and wrist at neutral position with the palm facing inwards.

## Data Availability

The full datasets related to this study are available on request from the corresponding author due to ethical reasons. The dataset and detailed statistical analysis outputs supporting the findings of this study are openly available on the Open Science Framework at https://osf.io/n4gv5 (DOI: 10.17605/OSF.IO/N4GV5).

## Abbreviations

The following abbreviations are used in this manuscript:

%BF	percentage of body fat
2D	index finger
2D:4D	the ratio of the length of the second digit (index finger) to the length of the fourth digit (ring finger) on a hand
4D	ring finger
ANOVA	analysis of variance
AP1	1st arm posture (shoulder at 0 deg flexion, elbow at 180 deg extension, wrist at neutral position with the palm facing towards the body)
AP2	2nd arm posture (shoulder at 0 deg flexion, elbow at 90 deg flexion, wrist at neutral position with the palm facing inwards)
AP3	3rd arm posture (shoulder at 180 deg flexion, elbow at 180 deg extension, wrist at neutral position with the palm facing inwards)
BMI	body mass index
ICC	intraclass correlation coefficient
LBM	lean body mass
LMM	linear mixed model
MIVC	maximum handgrip isometric voluntary contractions
PID	participant identification number
REML	restricted maximum likelihood
U16	under 16 years old age category
